# Extreme Ultraviolet to Visible Dispersed Single Photon Detection for Highly Sensitive Sensing of Fundamental Processes in Diverse Samples

**DOI:** 10.3390/ma11060869

**Published:** 2018-05-23

**Authors:** Andreas Hans, Philipp Schmidt, Christian Ozga, Gregor Hartmann, Xaver Holzapfel, Arno Ehresmann, André Knie

**Affiliations:** Center for Interdisciplinary Nanostructure Science and Technology (CINSaT), University of Kassel, Heinrich-Plett-Straße 40, 34305 Kassel, Germany; p.schmidt@uni-kassel.de (P.S.); ozga@physik.uni-kassel.de (C.O.); gregor.hartmann@physik.uni-kassel.de (G.H.); x.holzapfel@physik.uni-kassel.de (X.H.); ehresmann@physik.uni-kassel.de (A.E.)

**Keywords:** photon spectroscopy, fluorescence, single photon detection, atomic and molecular physics, clusters, synchrotron radiation

## Abstract

The detection of a single photon is the most sensitive method for sensing of photon emission. A common technique for single photon detection uses microchannel plate arrays combined with photocathodes and position sensitive anodes. Here, we report on the combination of such detectors with grating diffraction spectrometers, constituting a low-noise wavelength resolving photon spectroscopy apparatus with versatile applicability. We recapitulate the operation principle of such detectors and present the details of the experimental set-up, which we use to investigate fundamental mechanisms in atomic and molecular systems after excitation with tuneable synchrotron radiation. Extensions for time and polarization resolved measurements are described and examples of recent applications in current research are given.

## 1. Introduction

The detection of luminescence is one of the key techniques in experimental science. Photons emitted after diverse excitation schemes carry information on the electronic and atomic structure of matter, nature and lifetime of excited states, as well as on dynamic processes like dissociation, charge and energy transfer processes. Depending on the field of science and the actual application, the photon detection schemes vary substantially. In this article, we describe dispersed single photon counting from the extreme ultraviolet to visible spectral range using microchannel plate based detection. This technique is ideally suited to be applied in fundamental research, where the interaction of an excitation source with single objects (ions, atoms, molecules, clusters) in a dilute target is intended to be investigated.

In the following paragraph, we give an overview of our experimental set-up and the working principle of our single photon detectors. In Section 2, the components of these kinds of detectors and their properties are discussed in detail. The combination with diffraction photon spectrometers as an efficient configuration for dispersed emission measurements is described in Section 3. We give examples of our recent research and further applications of single photon spectroscopy in fundamental physics and physical chemistry in Section 4.

**Figure 1:**
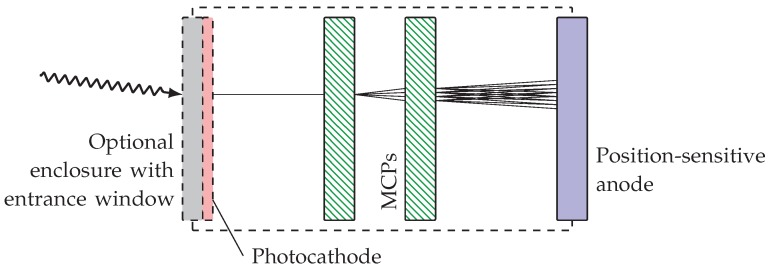
Illustration of the design of a single photon counting detector. Photons impinging on the (optional) photocathode (red) or the microchannel plates (MCP) (green) release photoelectrons, which are multiplied by MCPs. The resulting charge cloud is detected using a position sensitive anode (blue).

The design of a single photon detector based on a combination of microchannel plates (MCPs) and a position sensitive anode is schematically illustrated in Figure 1. Photons are either directly converted into electrons by the photoelectric effect in the top MCP or an additional photocathode with enhanced photoelectron yield is used to convert the photon into an electron. This single electron is multiplied by a stack of microchannel plates (MCPs) by a factor of up to $10^{7}$. The center of mass of the resulting charge cloud is detected spatially resolved by a position sensitive anode. This scheme is applied in a wide field of physics for single particle detection, including photons [1,2,3,4], because of its main advantage: the ultra low noise rate of few counts per second over the full detector area.

Different anode designs allow the position resolved counting of pulses and thereby the determination of the number of incident photons on a defined detector region. In combination with a dispersing optical element, the position on the detector encodes the photon wavelength. The counting of single photons enables the investigation of weak processes with unprecedented sensitivity. To record emission spectra over a wide spectral range is time consuming. Most times, only the simultaneous detection of sufficiently large spectral intervals makes such experiments possible—utilizing position sensitive detectors.

## 2. Detector Details

### 2.1. Photocathodes

In the scheme described here, the creation of a photoelectron is a prerequisite for the detection of an incident photon. The sensitivity of a detector to photons within a certain spectral range is therefore governed by the photoelectron yield of the material the photon impinges on. Usually, this capability is enhanced by using a suitable photocathode material. Since most of these photocathode materials are subject to oxidation under atmospheric conditions, it is deposited as a thin film on the rear side of a window substrate with respect to the photon incidence and the complete assembly, including MCPs, is encapsulated under vacuum. The choice of photocathode and window material strongly determines the sensitivity range of the detector. The spectral response, i.e., the probability that a photon of a certain wavelength λ is detected, depends on the transmission $T_{w}\left(\lambda \right)$ of the window substrate and the probability $P(\sigma_{\lambda },d_{PC})$ that the impinging photon releases a photoelectron. As indicated, *P* is a function of the (wavelength dependent) efficiency of the photoelectric effect of the photocathode material $\sigma_{\lambda }$ and the cathode thickness *d*. However, the photocathode must be a thin layer because the photon is absorbed on one side of the film and the photoelectron has to escape from the other side towards the front of the MCP array, as illustrated in Figure 1. A voltage is usually applied between photocathode and the first MCP to maintain the spatial coordination since photoelectrons can be emitted with transverse momentum.

For detection of photons with λ≲ 130 nm (corresponding to a photon energy of about 9.5 eV), no photocathode is required in general because the photon energy is sufficient to release photoelectrons from the conductive MCP coating itself. In addition, there is no substrate material in this spectral range with considerable transmission to obtain reasonable detection efficiencies. Photocathode materials may also be deposited directly on the MCP surface to alleviate the need of a transmitting window. In practice, however, such detectors have to be stored and operated under vacuum or inert gas conditions permanently to avoid oxidization of the cathode material.

**Figure 2:**
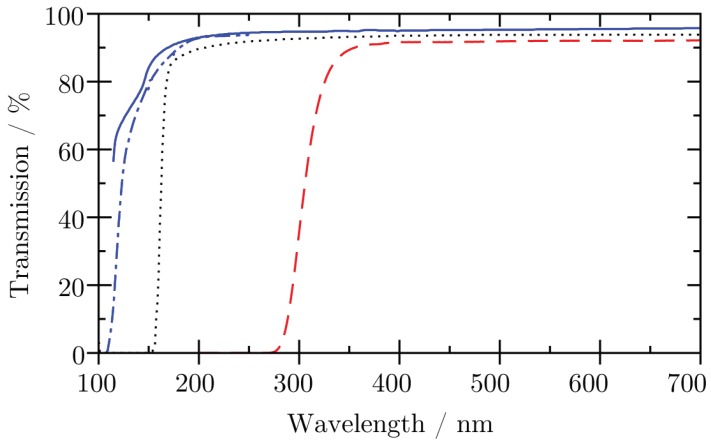
Transmission of three common window materials as a function of the photon wavelength as declared by the supplier. Blue solid line: MgF_2_ [5], blue dash-dotted line: MgF_2_ [6], black dotted line: fused silica (”suprasil”) [5], red dashed line: N-BK7 [7].

For λ> 130 nm, a photocathode is required and typically used materials for ultraviolet (UV) and visible photon detection are caesium telluride, caesium iodide, and multialkali coatings. Fluoride based materials such as LiF and MgF_2_ serve as the substrates with the lowest cut-off wavelength for transmission (depends on the surface roughness, optimally ≈110 nm can be reached) with further materials available for higher wavelengths, such as fused silica (≈160 nm). In Figure 2, the transmission of three common window materials is shown. Table 1 lists commonly used combinations of photocathode materials and window substrates, and the resulting sensitivity range. Note that, of course, there is no sharp limit of the sensitivity.

### 2.2. Microchannel Plates

Microchannel plates can be thought of as arrays of $10^{4}$–$10^{7}$ miniature electron amplification tubes. The diameter of each channel is typically between 6 and 12.5 microns, but can range up to some tens of microns. The channel length, which is approximately the thickness of the plate, varies from about hundred microns to about one millimeter. The aspect ratio *a* of channel length to diameter is an important characteristic determining the achievable amplification. The rear side of the plate is set to a high positive bias (typically between some hundred and approximately 1200 V) with respect to the front side. Impinging particles, namely photons with sufficient energy or electrons from a photocathode, release secondary electrons from the channel surface. These electrons are accelerated towards the MCP rear side, but will soon hit the channel wall because they have a velocity component perpendicular to the channel axis. Since they were accelerated, they have enough kinetic energy to release multiple secondary electrons from the channel surface. This process repeats until the electron cloud reaches the end of the channel. Typically, an amplification of $10^{4}$ per MCP can be reached. More than one MCP may be stacked in series until space charge effects prevent further amplification. The channel axis is usually tilted by an angle θ with respect to the plate surface by a few degrees to prevent so-called ion feedback. Here, residual gas cations are accelerated towards the front side of the MCP by the high voltage bias and would trigger additional photoelectrons if they hit the channel wall by the high voltage bias. Tilted channels force these ions to collide with the walls very close to the rear side, leading to electron amplification only below the detection threshold. If MCPs are stacked in series, the channel axes should be oriented opposite. Corresponding to the resulting pattern of the channel axes, stacks of two MCPs are typically called “chevron” and stacks of three MCPs “z-stacks”. The total gain achieved by an array of MCPs can, in general, be expressed as a function of *a*, θ, and the operating voltage $U_{HV}$. The characteristics and properties of MCPs are discussed in more detail in Refs. [8,9].

Because a high voltage is applied between front and rear side, the resistance of an MCP must be high and is typically in the order of 10 to 100 MΩ. All MCP based detectors have to be operated under high vacuum and may be damaged irreparably if the high voltage is applied under inadequate vacuum conditions. When stacked, MCP surfaces can be in contact or kept at a small distance. In the former case, the plates should be “matched”, which means they should have roughly the same resistance if the bias is applied between the front of the first MCP and the back of the last MCP to result in a homogenous voltage division. For some applications, it is desired to apply a bias between the rear side of the first MCP and the front of the second, and thus the additional space in between (ranging typically from 100 microns up to a few millimeters) is required. This way, more channels of the second MCP can be involved in the amplification process by choosing an appropriate bias.

The size of an MCP is typically ranging from a few millimeters up to several centimetres. The channel density ρ at the surface is an important quantity influencing the overall detection efficiency of the detector. Recently, MCPs with funnel-shaped channels have been developed to increase ρ [10]. An evenly distributed potential at each surface must be ensured by a conductive coating. Nevertheless, different detection regions can have different amplifications resulting in a spatially distributed detection threshold (see Section 3). Still, an essential advantage of MCP-based detection is the preservation of the information on the position of incidence of the detected particle throughout the amplification process. Beside simple discrimination between detector sections (e.g., to increase the signal-to-noise ratio), this can be used in detection schemes where the detected position carries physical information of the detected particle. To read out the position of incidence, the amplified electron cloud is detected on a position sensitive anode behind the MCPs.

An essential advantage of MCPs is the low noise compared to other photon imaging devices such as silicon photomultipliers or charge-coupled device (CCD) cameras. This ensures an acceptable signal-to-noise ratio for low real signal rates in the low Hz or even below Hz regime, particularly when the excitation source has a comparably large repetition rate. In these instances, CCDs require either long exposure times or frequent read-outs, which add significant amounts of noise.

### 2.3. Position Sensitive Anodes

The information of the position of a single event is conserved during the amplification process and the charge cloud leaves the MCP centered around the same (x,y) position where the initial electron was released by the impinging photon (*x* and *y* are here defined as the axes of a two-dimensional right-angled coordinate system). In order to retrieve the position information, position sensitive anodes are used. For optimal utilization, the active area of the anode should be at least of the same size as the MCPs. The principles of three commonly used anode types are described briefly in the following paragraphs.

#### 2.3.1. Wedge and Strip Anode

**Figure 3:**
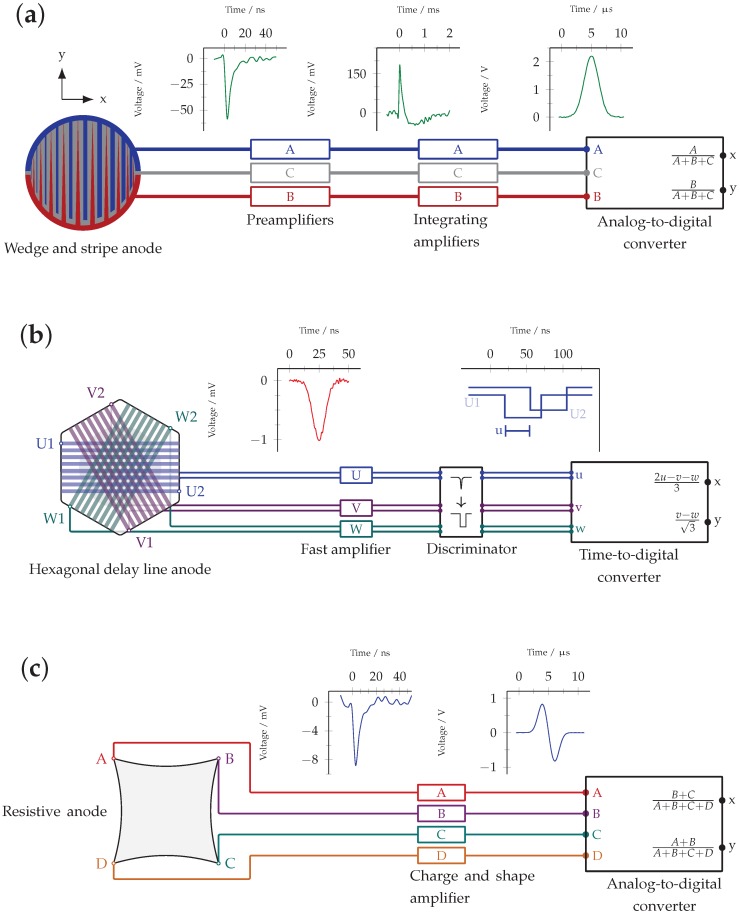
Operation principle and signal processing of the three used anode types. (**a**) wedge and strip anode: the position information is entailed in the amount of charge reaching the anode, i.e., the signal is processed through preamplifiers and integrating amplifiers and read out by an analog-to-digital converter (ADC). The shown coordinate system demonstrates the orientation of the *x* and *y* axis and is valid for all anode types. (**b**) delay line anode: the position is determined from signal delay times, the pulses are processed by fast amplifiers and read out by a time-to-digital converter (TDC); (**c**) resistive anode: the position is determined from signal heights at four anode edges at 45° with respect to the coordinate axes. Here, the signals are processed through amplifiers and a position computer and read out by an ADC.

With wedge and strip anodes, the position information is obtained by measuring the amount of charge (amplified to voltage pulses of different heights) reaching three different anode parts, each isolated with respect to each other [11]. The anode design and the signal processing is illustrated in Figure 3a. The anode area is divided in parts *A*, *B*, and *C*. Part *A* are strips, equally spaced, but with increasing width towards higher *x* values. *B* resembles equally spaced wedges along the *y*-direction, with their base at larger *y* and their apex at lower *y*. Part *C* is the area in between *A* and *B* and is called meander. If normalized to the sum A+B+C, the amplitudes of voltage pulses from *A* and *B* can be transferred into the position (x,y) by a transformation *f*:

(1) x=f(A/(A+B+C))y=f(B/(A+B+C)).

In explicit applications, it is typically sufficient to use a linear transformation for *f*. If, however, spatial imperfections shall be corrected to a very high degree, a transformation of higher order might be necessary. Typically, a resolution of 2% of the detector area can be achieved. Because the total amount of charge is the measure of interest using wedge and strip anodes, the raw anode pulses are amplified by an integrating amplifier and then read out by an analog-to-digital converter (ADC).

#### 2.3.2. Delay-Line Anodes

The working principle of delay line anodes is shown in Figure 3b. Here, the position information is obtained by measuring the arrival time differences of voltage pulses at two opposite ends of wires, spanned across the detector area. In principle, the (x,y) information can be retrieved from signals from four ends of two non-collinear oriented wires. The spatial resolution can, however, be improved if a set of three wires is used oriented in 60${}^{\circ }$ with respect to each other. The position information is then over-determined and typically computed as the mean value of the three positions given from different wire combinations. The position (x,y) can be calculated from the six arrival times of signals at the ends of the three wires U,V,W by the following relations [12]:

(2) $$x=\frac{2u-v-w}{3}\;y=\frac{v-w}{\sqrt{3}}.$$

The small letters u,v,w designate the delay time of the signals arriving at the ends of each wire, i.e., $u=U_{1}-U_{2}$, $v=V_{1}-V_{2}$, and $w=W_{1}-W_{2}$. Here, the coordinate system is defined with *x* parallel to *U*, *V* and *W* disposed by ± 120 degrees. Delay-line anodes provide resolution of about 0.5% of the detector area.

#### 2.3.3. Resistive Anodes

With resistive anodes, the signal height reaching four different points *A*, *B*, *C*, and *D* of one anode is recorded, which are located on the bisectrix of the (x,y) coordinate system [13]. They contain the position information by the transformation [14]

(3) $$x=\frac{B+C}{A+B+C+D}\;y=\frac{A+B}{A+B+C+D}.$$

The working principle of resistive anodes is shown in Figure 3c. Typically achievable resolutions of resistive anodes are about 0.5% of the detector area.

These three anode types are commonly used in MCP-based detector systems. A certain anode type might be suited best for a specific application, but, in principle, all types can be used for single photon counting as described here. While delay-line anodes usually perform best with respect to spatial and temporal resolution and are potentially capable of multi-hit detection, wedge and strip anodes and resistive anodes can be operated using somewhat simpler electronics and data processing.

In explicit applications, it is typically sufficient to use a linear transformation for *f*. If, however, spatial imperfections shall be corrected to a very high degree, a transformation of higher order might be necessary. Typically, a resolution of 2% of the detector area can be achieved. Because the total amount of charge is the measure of interest using wedge and strip anodes, the raw anode pulses are amplified by an integrating amplifier and then read out by an analog-to-digital converter (ADC).

#### 2.3.4. Delay-Line Anodes

The working principle of delay line anodes is shown in Figure 3b. Here, the position information is obtained by measuring the arrival time differences of voltage pulses at two opposite ends of wires, spanned across the detector area. In principle, the (x,y) information can be retrieved from signals from four ends of two non-collinear oriented wires. The spatial resolution can, however, be improved if a set of three wires is used oriented in 60${}^{\circ }$ with respect to each other. The position information is then over-determined and typically computed as the mean value of the three positions given from different wire combinations. The position (x,y) can be calculated from the six arrival times of signals at the ends of the three wires U,V,W by the following relations [12]:

(4) $$x=\frac{2u-v-w}{3}\;y=\frac{v-w}{\sqrt{3}}.$$

The small letters u,v,w designate the delay time of the signals arriving at the ends of each wire, i.e., $u=U_{1}-U_{2}$, $v=V_{1}-V_{2}$, and $w=W_{1}-W_{2}$. Here, the coordinate system is defined with *x* parallel to *U*, *V* and *W* disposed by ±120 degrees. Delay-line anodes provide resolution of about 0.5% of the detector area.

#### 2.3.5. Resistive Anodes

With resistive anodes, the signal height reaching four different points *A*, *B*, *C*, and *D* of one anode is recorded, which are located on the bisectrix of the (x,y) coordinate system [13]. They contain the position information by the transformation [14]

(5) $$x=\frac{B+C}{A+B+C+D}\;y=\frac{A+B}{A+B+C+D}.$$

The working principle of resistive anodes is shown in Figure 3c. Typically achievable resolutions of resistive anodes are about 0.5% of the detector area.

These three anode types are commonly used in MCP-based detector systems. A certain anode type might be suited best for a specific application, but, in principle, all types can be used for single photon counting as described here. While delay-line anodes usually perform best with respect to spatial and temporal resolution and are potentially capable of multi-hit detection, wedge and strip anodes and resistive anodes can be operated using somewhat simpler electronics and data processing.

### 2.4. Time Resolved Detection

**Figure 4:**
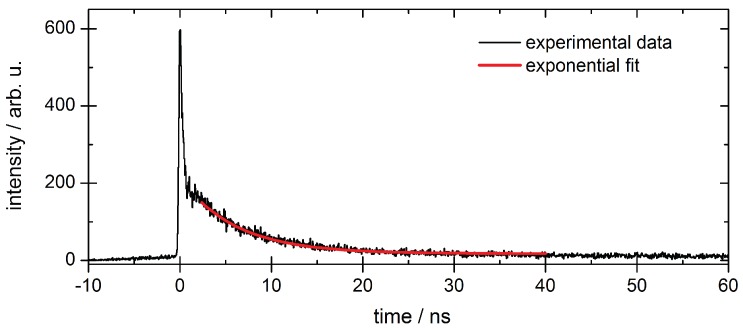
Example for a histogram of event times relative to the exciting synchrotron radiation pulse (set to time zero). The lifetime is given by the decay constant τ in an exponential decay process of the form $y=y_{0}+A_{0}\cdot e^{-\frac{t}{\tau }}$, with offset $y_{0}$ and scaling factor $A_{0}$. The example shows the radiative decay in the vacuum ultraviolet range after photoexcitation of the 1s→3p resonance in neon. The exponential fit yields an integrated lifetime of τ=(6.2±0.2) ns in this spectral range. The sharp peak at t=0 appears due to detection of scattered synchrotron radiation and is therefore excluded from the fit.

If the source of excitation is pulsed, MCP based detectors offer the possibility of time resolved photon detection. The typical time resolution of MCPs of about 50 ps allows the measurement of lifetimes of most radiative states, which are usually on the nanosecond time scale. A weak voltage pulse can be coupled out capacitively from the short breakdown of the high voltage supply of the MCP when an event is detected. To explicitly determine the lifetime of a radiatively decaying state, the time between excitation and detection has to be measured for each event and all such obtained event times are listed in a histogram. If the exciting pulse is short compared to the decay, the lifetime can be extracted by fitting an exponential decay. The time distance between subsequent excitation pulses is required to be sufficiently long compared to the lifetime of the decaying state observed by photon emission. If both are within the same order of magnitude, the lifetime can still be estimated by considering the signal pile up from several pulses. As an example, in Figure 4, the event time histogram of a radiative decay in neon is shown.

Time resolved event detection also enables the application of coincidence techniques. We developed an apparatus for photon-photon coincidence experiments [15]. However, coincidence experiments using photon detection are extremely challenging, since the solid angle cannot be enlarged by applying electric and magnetic fields as for charged particles (see e.g., [16,17]), and reports of successful experiments are scarce [18,19,20].

### 2.5. Detector Efficiency

The total detector efficiency Δ, i.e., the probability that a photon impinging on the detector is recorded as an actual event by the data acquisitioning system, is given by the product of the efficiencies of its components:

(6) $$\Delta =T_{w}\left(\lambda \right)\cdot P\left(\sigma_{\lambda },d_{PC}\right)\cdot \rho \cdot \eta .$$

**Figure 5:**
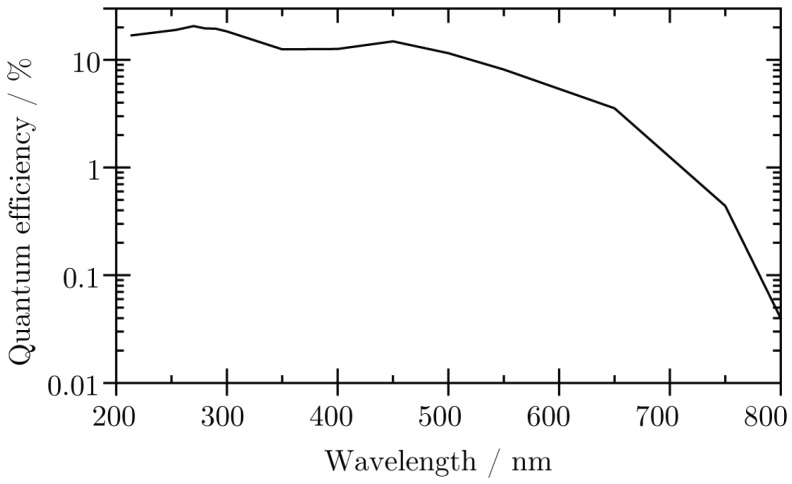
Exemplary detector efficiency as function of photon wavelength of a *Photek MCP240* detector with a fused silica window and bialkali photocathode [21].

The quantity η is introduced to account for the signal processing after the photocathode. It includes the gain of the MCP array, which is a function of the channel aspect ratio and the operating voltage (see Section 2.2). The charge cloud reaching the anode is further processed as electrical signal, depending on the anode type and according to Figure 3. The detection probability of an anode signal depends mainly on the noise level and processing thresholds of electronic devices. There is some loss of events, for which the amplification of the MCPs does not reach the processing thresholds, i.e., the lower tail of the pulse height distribution. Typical pulse height distributions are discussed in detail in Refs. [8,9]. In Figure 5, an exemplary detector efficiency as a function of the photon wavelength of a commercial *Photek MCP240* detector system (St Leonards-on-Sea, UK), as it is used in our set-up, is shown. This detector is equipped with a bialkali photocathode deposited on a fused silica window substrate.

### 2.6. Spatial Effects

The detector efficiency Δ, i.e., the probability that a photon impinging on the detector is recorded as an actual event, as well as the spatial resolution is not constant across the detector area. This is, due to manufacturing imperfections of MCPs, voltage supply connection, and anode, an intrinsic property of the detector. Furthermore, MCPs and photocathodes show aging effects, if certain areas are stressed more than others. Therefore, it is necessary to characterize the spatial response Δ(x,y,λ) and scale the measured data accordingly to deduce conclusions from relative intensities of features detected on different detector regions.

## 3. Combination of Single Photon Detection with Spectrometers

### 3.1. Spectrometer–Detector Combination

The technique of position resolved photon detection is especially powerful if combined with photon spectrometers. In such a spectrometer, an optical grating is typically used to disperse and thereby separate photons of different wavelengths along an axis. If no spatial detection is available, emission spectra must be recorded by monochromating the dispersed light with an exit slit and detecting the intensity by scanning the selected wavelength. Using position sensitive detection, however, a partial spectrum can be detected at once with its range depending on the magnitude of dispersion and the active detector area. This way, the time required for recording emission spectra is reduced drastically.

**Figure 6:**
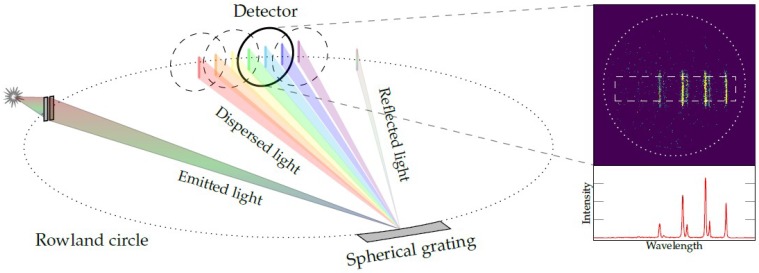
Illustration of the combination of a photon spectrometer with position sensitive detection. Light emitted in a source volume enters the spectrometer through an entrance slit and is dispersed by a spherical grating. In reality, the detector is fixed in space and the grating is moved to project different spectral ranges onto the detector. The inset shows an exemplary detector image, from which the emission spectrum is obtained by integrating the signal within a region of interest (dashed rectangle) over the vertical coordinate.

The grating and the detector are mostly used in the Rowland geometry [22]. Here, a spherical reflection grating with surface curvature of radius 2R disperses and projects light from a source volume lying on the Rowland circle with radius *R* back onto the circle, as illustrated in Figure 6. To minimize imaging errors, the detector is mounted tangentially to the Rowland circle. The source volume is given by the interaction volume of the excitation source with the target or, in special cases, the spectrometer entrance slit. From the recorded detector image, the partial emission spectrum is obtained by intensity integration over the direction perpendicular to the dispersion axis, and this is displayed in the inset of Figure 6.

The properties of the optical grating determine the dispersion magnitude and the spectral range with highest reflectivity and should be chosen to match the sensitivity range of the detector. For enhancing the reflectivity in a certain spectral range, the grating is usually coated and blazed. The latter means that the grating surface is tilted locally with respect to the surface normal, thereby increasing the reflectivity under a certain angle. The fraction of photons Λ reaching the detector after being emitted in a source volume can be expressed as

(7) $$\Lambda =\Omega \left(L,A_{G},d_{slit}\right)\cdot \xi \left(\beta \right)\cdot T_{E}\left(\lambda \right)\cdot R_{G}\left(n,\lambda ,\lambda_{blaze},\mathrm{coating}\right).$$

Here, Ω is the solid angle, which depends on the distance *L* between source volume and grating, the grating area $A_{G}$, and possibly the entrance slit width $d_{slit}$. ξ(β) is a factor accounting for non-isotropic radiation from the target, which depends on the anisotropy parameter β of the observed process (see Section 3.2). The parameter $T_{E}\left(\lambda \right)$ represents the transmission function of any window material in the path from source volume to grating (see Section 4.1). The reflection properties of the grating are included in factor $R_{G}$, depending on the photon wavelength λ, the order *n* of diffraction, the blaze wavelength $\lambda_{blaze}$, and the grating coating. Finally, the detection efficiency *Q* of the whole set-up, i.e., the probability that a photon emitted from a source volume is detected as an event is given by

(8) Q=Λ·Δ·η.

The resolving power of the apparatus depends on several parameters—firstly, by the width of the source volume or entrance slit to the spectrometer, which is projected onto the detector and determines the apparative width of spectral lines (The entrance slit is still desirable if the actual source is even thinner than the slit itself, e.g., a focused photon beam, to prevent stray light entering the spectrometer.); secondly, by the line density of the grating. The resolving power is proportional to the line density and the order of diffraction. However, with increasing line density the absolute range of the partial spectrum which fits onto the detector area is narrowed. Thirdly, by the resolving power of the detector itself. Depending on the application, the combination of the three mentioned parameters should be optimized for count rate and time efficient measurement or highest resolution. If optimized for, typically a resolution of about 1 Å can be achieved.

To record emission spectra in a wider emission wavelength range, the grating, and hence the Rowland circle, may be reoriented inside the chamber. By varying the direction with respect to the incident photons, the partial spectrum on the detector area can be chosen. The full spectrum in a range of interest can be obtained by measuring several partial spectra subsequently and merging them afterwards. For this procedure, it is recommended to record the partial spectra with sufficient overlap to identify the correct offset when merging.

In general, the emission spectrum wavelength axis is a function of the arbitrary projection the data acquisitioning system generates when reading out the position. A coarse calibration of the wavelength axis can be achieved by the position of the zeroth diffraction order, i.e., the direct reflection (which is per definition at zero emission wavelength, see Figure 6) and the position of the grating (which is typically displayed at the spectrometer). A fine calibration can be realized by identifying the position of known emission lines and fitting a linear function which transforms the axis from arbitrary units to absolute wavelengths. If no transition from a target of interest is known, a separate calibration with a known target might be necessary.

### 3.2. Measurement of Angular Distributions

The angular distribution of photon emission often contains important information on the nature of decaying states and interference effects between competing channels. Typically, the anisotropy parameter β is used to quantify the angular distribution [23]. In principle, there are two ways to determine it experimentally. Firstly, the photon emission may be measured under two different emission angles to fit β from the relative intensities. Contrary, the second possibility is to measure only in one emission direction but resolving the photon polarization. It can be shown that both methods yield the same information [24].

The first method requires the operation of two comparable spectrometer-detector combinations for the same spectral range under different emission angles simultaneously, or, alternatively turning of the polarization vector of the exciting photon beam. Polarization resolved detection can be realized by mounting a Wollaston prism in the optical path of the photons between the grating and the detector. The Wollaston prism separates parallel and perpendicular polarized light spatially, which then reaches different areas on the detector. In several works, we used this method to determine β experimentally [25,26,27]. It should be noted that the configuration with two spectrometers can also be used to detect photons in two different spectral ranges simultaneously, as it was applied in Ref. [28], possibly with polarization resolving Wollaston prism in one or both spectrometers.

## 4. Examples of Applications

The focus of our work is the electronic structure of quantum systems and decay processes after the excitation of matter by photon or electron impact. We use light of energy in the order of the binding energies of electrons to address electronic excitations, which typically governs an energy range from the ultraviolet at a few electron volts (eV) up to the soft X-ray regime of several hundred eV. The source of choice for photons in this energy range is synchrotron radiation, which offers complete tunability and surpasses other light sources by orders of magnitude with respect to photon flux and bandwidth [29]. In the following we give a few examples of our recent research using single photon detection from different targets after excitation with synchrotron radiation. We emphasize, however, that the detection principle is not constraint to be applied in combination with this excitation source.

### 4.1. Target Delivery and Differential Pumping

Depending on the sample of interest, different target delivery systems can be used in combination with the photon spectroscopy set-up. For gaseous samples, typically a gas cell is used. The cell is mounted inside the interaction chamber and has a constant gas flow provided from outside the vacuum chamber. By using flow regulating valves, the pressure inside the chamber can be adjusted and kept constant. The only openings of the cell are apertures in the order of few millimeters as entrance and exit for the exciting radiation. If photons beyond the vacuum ultraviolet spectral range are detected, a narrow slit towards the spectrometer is used. For detection in all spectral ranges with longer wavelengths than about 120 nm, window materials (see Section 2) may be used towards the spectrometer and any further opening at which photons shall be detected. The usage of a cell with apertures drastically reduces the amount of gas that has to be removed from the interaction chamber by vacuum pumps in order to maintain suitable vacuum conditions and is further reduced if windows are used. Typically, pressures up to 1 mbar can be used in the gas cell.

For experiments involving weakly bound clusters gas cells are not a viable option. The formation of clusters can be achieved by supersonic expansion of gases with high pressure (0.1–10 bar) through a nozzle (diameter 10–200 μm) into the vacuum [30]. Because a cluster jet might be disturbed by any close objects, it should ideally travel freely through the chamber, excluding the use of a small cell with apertures. Under typical experimental conditions with target densities required for luminescence measurements, the pressure within the interaction chamber will be in the orders from $10^{-6}$ to $10^{-4}$ mbar.

Liquid samples can be provided using the liquid microjet technique [31]. Here, a liquid is injected under high pressure (2–20 bar) through a thin capillary (10–30 μm) into the vacuum. To reach proper vacuum conditions, the jet itself and gas evaporating from its surface must be frozen out on cryogenic surfaces, like liquid nitrogen filled cryo traps. In such experiments, the typical pressure in the interaction chamber is in the range of $10^{-4}$ mbar.

For each sample, the pressure within the interaction chamber is far above the pressure acceptable for many sources of electronic excitation, especially for vacuum ultraviolet (VUV) or soft X-ray light provided by synchrotron radiation facilities. A differential pumping section is placed between the chamber and the source to compensate several orders of magnitude in pressure [32]. Different implementations for differential pumping sections are common, depending on the required final pressure and the amount of gas or pressure in the interaction chamber with operating target source. In principle, it consists of a sequence of chambers, each separated by apertures and equipped with turbomolecular pumps. Typically, a three-stage section is used that can maintain a $10^{3}$–$10^{4}$ pressure ratio resulting in final pressure in the order of $10^{-9}$ mbar.

### 4.2. Gas Phase Targets

The investigation of gaseous atomic and molecular species is essential for the exploration of fundamental light–matter interaction and to provide benchmark data for theoretical models. The detection of luminescence from photoexcited molecules and from excited products of photodissociation enables the determination of relative, and in some cases absolute, production cross sections of molecules or fragments in distinct quantum states because photon emission is both species and state selective.

**Figure 7:**
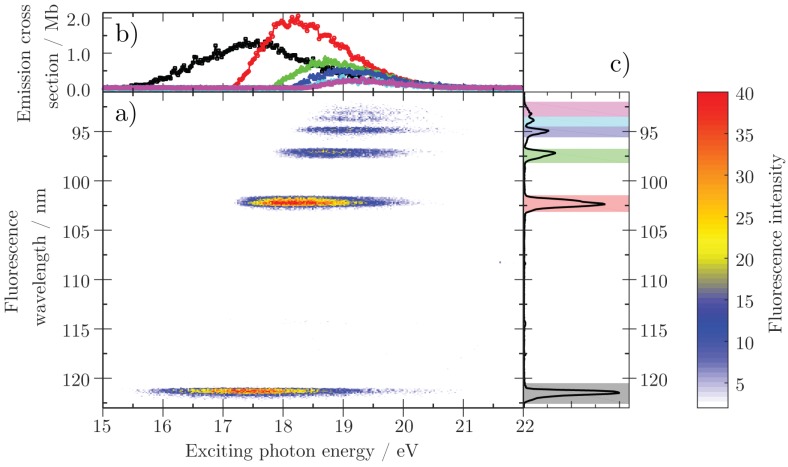
Absolute emission cross section of single transitions of the Lyman series emitted by neutral hydrogen fragments after photodissociation of valence excited water molecules (data taken from [36]). (**a**) 2D colormap of the emission intensity of the Lyman transitions as function of the exciting photon energy; (**b**) absolute emission cross section of the transitions $Ly_{\alpha }$ to $Ly_{\epsilon }$ as function of the exciting photon energy. For calibration procedure see Ref. [36]; (**c**) emission spectrum integrated over exciting photon energy. Each Lyman transition is colored according to the emission cross section graphs in panel (**b**).

This method is especially powerful if combined with tuneable excitation sources, e.g., synchrotron radiation sources, because the dynamics of molecules can then be investigated as a function of the exciting photon energy, adding state selectivity in the excitation process as well. The photodissociation of small molecules has been investigated extensively over the past decades by photon-induced fluorescence spectroscopy, focusing mostly on gaseous di- and triatomic species like H_2_ [33], N_2_ [34], O_2_, NO [23], N_2_O [35], and CO [26]. More recently, we determined the absolute production probability of excited hydrogen fragments after valence photoexcitation of the water molecule H_2_O [36]. The main result of this work is shown in Figure 7. By measuring the Lyman series emission from a neutral hydrogen dissociation fragment, the dissociation cross section into excited hydrogen fragments with different quantum number *n* was quantified. In addition, the investigation of atomic species can reveal relative or absolute cross sections of state selective photoionization and autoionization processes, as it was demonstrated mostly for noble gas targets (see e.g., [28,32,37,38,39]).

### 4.3. Cluster Targets

The physics of clusters has been of interest for a long time, as they serve as a prototypical bridge between single, isolated atoms or molecules and condensed bulk material [40,41]. Luminescence detection has been used extensively as a tool to characterize the development of the electronic structure from single atoms to clusters of various size to bulk matter [42].

During the last two decades, weakly bound clusters, like van der Waals or hydrogen bound systems, have attracted considerably attention due to the discovery of several non-local autoionization mechanisms. Since hydrogen bound clusters are considered to be prototype systems for biological systems, the reaction products of such autoionization mechanisms are believed to play a role in radiation biology. Photon detection as a complement to charged particle spectroscopy is applicable to neutral particles or dense media that do not transmit charged particles, as is the case for biological tissue.

**Figure 8:**
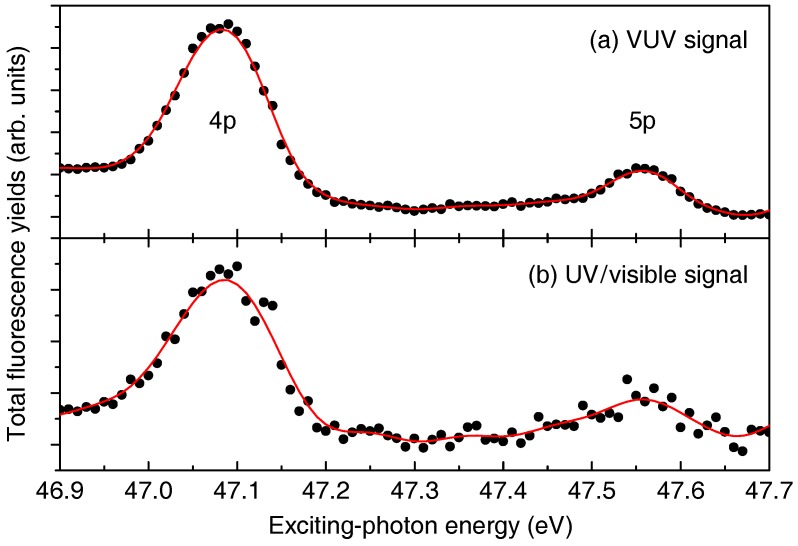
Partial photon yield excitation function in (**a**) the VUV (<130 nm) and (**b**) the UV/visible (300–630 nm) spectral ranges after 2s→4p and 2s→5p resonant excitation of neon clusters. The maxima appear due to the non-local autoionization process termed resonant interatomic Coulombic decay. Reprinted from *Chemical Physics* 482, 165–168, Copyright (2017) [44], with permission from Elsevier.

In a proof-of-principle experiment, we demonstrated that such interatomic non-local autoionization mechanisms can be detected by luminescence by choosing a showcase example, namely the resonant interatomic Coulombic decay (rICD) in neon clusters. The details of this mechanism are discussed in Ref. [27]. Briefly, it is ideally suited to be detected by luminescence since it opens a radiative decay channel after resonant 2s→*n*p excitation in a neon cluster, which is not present in isolated neon atoms. In Figure 8, the partial luminescence excitation function in the VUV and in the UV/visible spectral range after 2s→4p and 2s→5p resonant excitation of neon clusters is shown. The similarity of both signals can be explained by radiative cascades from rICD final states. In related works, it could also be shown that emission from clusters and atoms may be separated efficiently by time resolved measurements [27] and simultaneous observation of atomic and cluster emission enables an estimation of absolute cross sections of cluster-specific decay channels [43].

### 4.4. Liquid Targets

**Figure 9:**
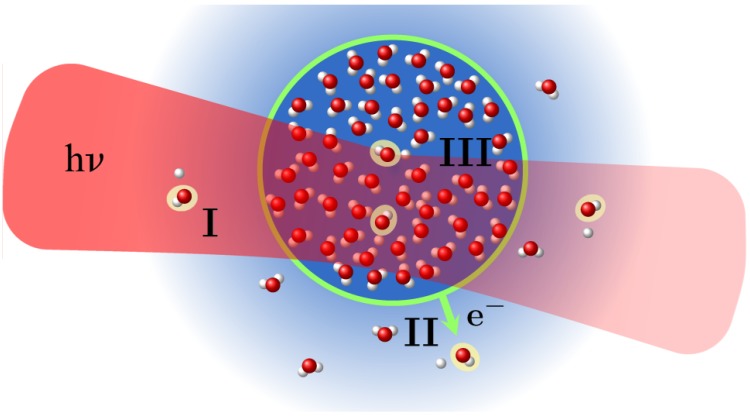
Illustration of a transversal section through a liquid microjet and indication of three processes (labelled I, II, and III) observed by photon spectroscopy after X-ray irradiation. For details see text and Ref. [45]. Reprinted with permission from *The Journal of Physical Chemistry* 121, 2326. Copyright 2017 American Chemical Society.

**Figure 10:**
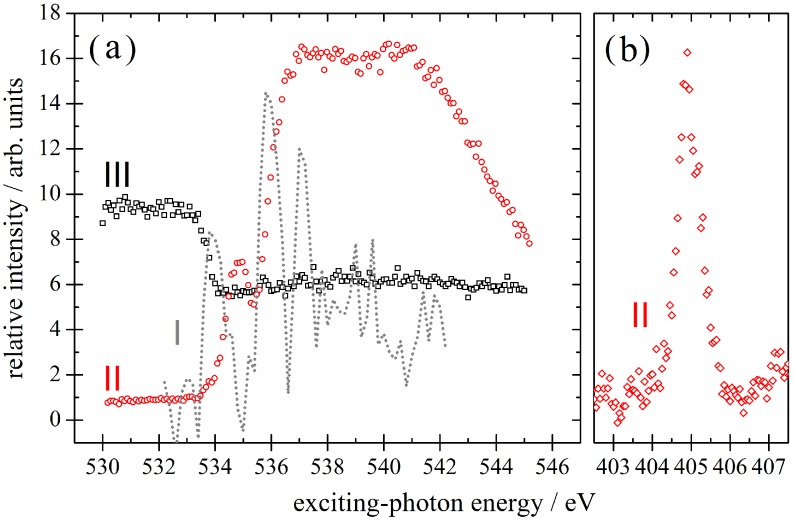
(**a**) emission intensity of three distinct processes initiated by the interaction of synchrotron radiation with a liquid microjet as a function of the exciting photon energy in the vicinity of the water *K*-shell threshold. The emissions are labelled according to the processes described in Figure 9; (**b**) intensity of emission following process II of a 1 molar NaNO_3_ solution in the vicinity of the nitrogen K-edge. For details, see text and Ref. [45]. Reprinted with permission from *The Journal of Physical Chemistry* 121, 2326. Copyright 2017 American Chemical Society.

The combination of a liquid microjet with a luminescence spectrometer opens the unique possibility to observe the response of the inner bulk of a liquid to X-ray irradiation. This region is inaccessible to electron spectroscopy due to the short mean free path of charged particles in this dense medium. Experiments on neat liquid water revealed the occurrence of three distinct pathways leading to photon emission: (I) the excitation of molecular water in the gaseous halo of the jet by direct irradiation with X-rays; (II) the excitation of molecular water in the gaseous halo of the jet by impact of fast electrons escaping from the jet-vacuum interface; and (III) extremely broad emission bands from the bulk liquid. Processes II and III have not been observed by other techniques before. All three processes are illustrated in Figure 9 [45]. In Figure 10, the emission intensity of these distinct processes are shown as a function of the exciting photon energy for pure water (a) and a 1 molar NaNO_3_ solution (b) in the vicinity of the water and nitrogen K-shell edge, respectively. By comparing the intensity behavior across the edges, the emission can be assigned to the different processes. For details, see Ref. [45].

## 5. Conclusions

We showed that single photon detection is a very versatile tool to track fundamental light matter interaction in various sample environments. The MCP based detection strategy allows to record nearly background free detection of the weakest signals, especially in combination with photon spectrometers. The method is not limited to the synchrotron radiation based experiments presented here, but can be advanced to any excitation scheme and sample delivery. Moreover, as the detection of photons does not require any applied fields, it can be readily combined to other spectroscopic methods, like electron or ion detection.

## Figures and Tables

**Table 1:** Common combinations of window and photocathode materials and their sensitivity range. The expression ”bialkali” usually refers to Sb-Rb-Cs or Sb-K-Cs materials and ”multialkali” (also ”S-20”) to Na-K-Sb-Cs material.

Window Material	Photocathode	Sensitivity Range
none	none, bare MCP	<130 nm
MgF_2_	none, bare MCP	115 nm to 130 nm
MgF_2_	CsTe	115 nm to 300 nm
MgF_2_	CsI	115 nm to 200 nm
MgF_2_	bialkali	115 nm to 650 nm
fused silica	multialkali, S-20	160 nm to 800 nm

## Abbreviations

The following abbreviations are used in this manuscript:

UV	ultraviolet
VUV	vacuum ultraviolet
MCP	microchannel plate
