# Erratum: Hans, A.; Schmidt, P.; Ozga, C.; Hartmann, G.; Holzapfel, X.; Ehresmann, A.; Knie, A. Extreme Ultraviolet to Visible Dispersed Single Photon Detection for Highly Sensitive Sensing of Fundamental Processes in Diverse Samples. *Materials* 2018, *11*, 869

**DOI:** 10.3390/ma12010066

**Published:** 2018-12-25

**Authors:** Andreas Hans, Philipp Schmidt, Christian Ozga, Gregor Hartmann, Xaver Holzapfel, Arno Ehresmann, André Knie

**Affiliations:** Center for Interdisciplinary Nanostructure Science and Technology (CINSaT), University of Kassel, Heinrich-Plett-Straße 40, 34305 Kassel, Germany; p.schmidt@uni-kassel.de (P.S.); ozga@physik.uni-kassel.de (C.O.); gregor.hartmann@physik.uni-kassel.de (G.H.); x.holzapfel@physik.uni-kassel.de (X.H.); ehresmann@physik.uni-kassel.de (A.E.)

The authors are sorry to report that the content between Figure 3 and the title *2.4. Time-Resolved Detection* of the published paper [1] is duplicated. Please delete it. The authors would like to apologize for any inconvenience caused to the readers by these changes.

The correct content is provided below:

**Figure 3.** Operation principle and signal processing of the three used anode types. (**a**) Wedge and strip anode: the position information is entailed in the amount of charge reaching the anode, i.e., the signal is processed through preamplifiers and integrating amplifiers and read out by an analog-to-digital converter (ADC). The shown coordinate system demonstrates the orientation of the x- and y-axis and is valid for all anode types; (**b**) delay line anode: the position is determined from signal delay times, the pulses are processed by fast amplifiers and read out by a time-to-digital converter (TDC); (**c**) resistive anode: the position is determined from signal heights at four anode edges at 45° with respect to the coordinate axes. Here, the signals are processed through amplifiers and a position computer and read out by an ADC.

*2.4. Time-Resolved Detection*
